# Identification of *Mycobacterium tuberculosis*-Specific Th1, Th17 and Th22 Cells Using the Expression of CD40L in Tuberculous Pleurisy

**DOI:** 10.1371/journal.pone.0020165

**Published:** 2011-05-18

**Authors:** Li Li, Dan Qiao, Xiaoying Fu, Suihua Lao, Xianlan Zhang, Changyou Wu

**Affiliations:** 1 Institute of Immunology, Zhongshan School of Medicine, Key Laboratory of Tropical Disease Control Research of Ministry of Education, Sun Yat-sen University, Guangzhou, People's Republic of China; 2 Chest Hospital of Guangzhou, Guangzhou, People's Republic of China; French National Centre for Scientific Research, France

## Abstract

Important advances have been made in the immunodiagnosis of tuberculosis (TB) based on the detection of *Mycobacterium tuberculosis* (MTB)-specific T cells. However, the sensitivity and specificity of the immunological approach are relatively low because there are no specific markers for antigen-specific Th cells, and some of the Th cells that do not produce cytokines can be overlooked using this approach. In this study, we found that MTB-specific peptides of ESAT-6/CFP-10 can stimulate the expression of CD40L specifically in CD4^+^ T cells but not other cells from pleural fluid cells (PFCs) in patients with tuberculous pleurisy (TBP). CD4^+^CD40L^+^ but not CD4^+^CD40L^−^ T cells express IFN-γ, IL-2, TNF-α, IL-17 or IL-22 after stimulation with MTB-specific peptides. In addition, CD4^+^CD40L^+^ T cells were found to be mostly polyfunctional T cells that simultaneously produce IFN-γ, IL-2 and TNF-α and display an effector or effector memory phenotype (CD45RA^−^CD45RO^+^CCR7^−^CD62L^−^ICOS^−^). To determine the specificity of CD4^+^CD40L^+^ T cells, we incubated PFCs with ESTA-6/CFP-10 peptides and sorted live CD4^+^CD40L^+^ and CD4^+^CD40L^−^ T cells by flow cytometry. We further demonstrated that sorted CD4^+^CD40L^+^, but not CD4^+^CD40L^−^ fractions, principally produced IFN-γ, IL-2, TNF-α, IL-17 and IL-22 following restimulation with ESTA-6/CFP-10 peptides. Taken together, our data indicate that the expression of CD40L on MTB-specific CD4^+^ T cells could be a good marker for the evaluation and isolation of MTB-specific Th cells and might also be useful in the diagnosis of TB.

## Introduction

Tuberculous pleurisy (TBP)—the second most frequent manifestation of extrapulmonary tuberculosis (TB) after lymph node TB—is restricted to the pleural cavity, which contains numerous immunocompetent cells [1]. TBP resolves spontaneously in some patients without treatment. Thus, TBP is thought to be a good model system for studying the protective immune response at the site of infection [2]. It is well recognized that cell-mediated immunity is required for an effective response to *Mycobacterium tuberculosis* (*MTB*) infection. In *MTB* infection, the Th1 response has been shown to be protective [3]. IFN-γ, a Th1 type cytokine that is the most important factor for macrophage activation, is essential for TB immunity [4]. Although some studies have shown that IL-4 is not essential for the development of a protective immune response to *MTB* [5], [6], Sugawara et al. showed that IL-4 is required for proper defense against TB infection [7]. In addition, Th17 cells can recruit granulocytes to the site of *MTB* persistence to produce inflammation [8]; these cells seem to play an important role in the early development of protective immunity in the lungs [8], [9], [10]. IL-22-producing CD4^+^ T cells are distinct from Th1 and Th17 cells and have also been shown to play important roles in the human immune response to mycobacteria [11].

Currently, the analysis and isolation of distinct antigen-specific Th cells relies on the detection of cytokines that are produced by these cells or the rare selection of specific multimers of peptide and major histocompatibility complex class ⨿ molecules. However, using cytokines to detect Th cells could bias the measurement of the frequencies of antigen-specific Th cells. Moreover, antigen-specific T cells that do not produce cytokines would be missed using this approach [12], [13], [14], [15]. Previous work has shown that the expression of CD40L (CD154) can be used to detect Th cells that are specific for defined antigens and to isolate viable Th cells for further study [16], [17]. CD40L is expressed by antigen-specific T cells following activation and could provide costimulatory signals to APCs (antigen-presenting cells) [18] and B cells [19], [20]. Because CD40L is only transiently expressed on the cell surface, it is difficult to detect [21], [22]. A modified assay has been developed that stabilizes the intracellular expression of CD40L with the secretion inhibitor Brefeldin A (BFA) [16]. Another assay uses the detection antibody and a protein transport inhibitor, monensin, to detect surface expression of CD40L directly [17].

In the present study, we demonstrated for the first time that the expression of CD40L on *MTB*-specific Th cells can be a good marker for the isolation of Th1, Th17 and Th22 cells. Furthermore, we found that CD40L expression on CD4^+^ T cells can be used to diagnose TB and for functional studies of *MTB*-specific Th cells.

## Results

### MTB-specific peptides induce the expression of CD40L by CD4^+^ T cells

**Figure 1:**
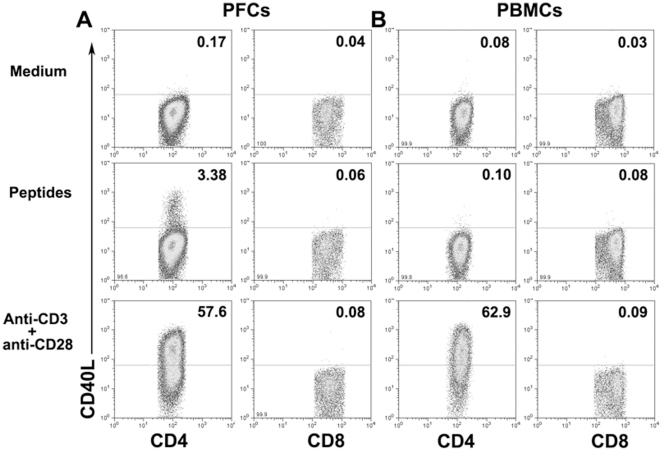
*M.tuberculosis* (MTB)-specific expression of CD40L on CD4^+^ and CD8^+^ T cells from pleural fluid cells (PFCs) of tuberculous pleurisy (TBP) and PBMCs from healthy individuals. PFCs from TBP and PBMCs from healthy donors were incubated for eight hours in the presence of medium alone, ESAT-6/CFP-10 peptides with anti-CD28 plus anti-CD49d or anti-CD3 plus anti-CD28 monoclonal antibodies (mAbs).(A) Expression of CD40L on CD4^+^ and CD8^+^ T cells in PFCs from TBP and (B) PBMCs from healthy donors were detected by FACS. Data shown are representative of five independent experiments.

We first investigated the specificity of CD40L expression by CD4^+^ T cells. Pleural fluid cells (PFCs) from tuberculous pleurisy (TBP) or PBMCs from healthy individuals were stimulated in the presence or absence of *MTB*-specific peptides (ESAT-6/CFP-10). As expected, we found that unstimulated cells do not express CD40L. In addition, CD40L expression was not detected in either CD4^+^ or CD8^+^ T cells of PBMCs following stimulation with ESAT-6/CFP-10 peptides (Fig. 1A). In contrast, CD40L expression could be clearly detected in CD4^+^ but not CD8^+^ T cells of PFCs following stimulation with ESAT-6/CFP-10 peptides (Fig. 1B). Statistical results indicated that significantly higher expression of CD40L by CD4^+^ T cells was induced in PFCs than PBMCs (data not shown), suggesting that CD40L expression is induced on antigen-specific CD4^+^T cells. However, no detectable expression of CD40L by CD8^+^ T cells from either PBMCs or PFCs was observed following either antigen-specific or polyclonal stimulation with anti-CD3 plus anti-CD28 (Fig. 1), suggesting that the expression of CD40L is predominantly restricted to CD4^+^ T cells.

### CD4^+^CD40L^+^ T cells primarily express IFN-γ, IL-2, TNF-α, IL-17 and IL-22

**Figure 2:**
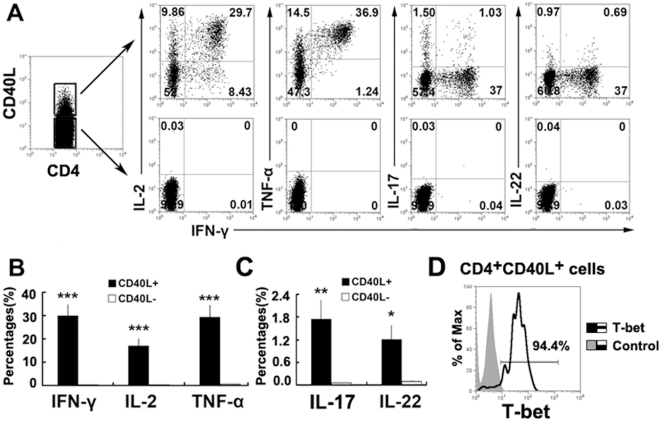
CD4^+^CD40L^+^ but not CD4^+^CD40L^−^ T cells express IFN-γ, IL-2, TNF-α, IL-17 or IL-22 following MTB-specific stimulation. PFCs were stimulated with ESAT-6/CFP10 peptides, anti-CD28 and anti-CD49d mAbs. CD4^+^CD40L^+^ and CD4^+^CD40L^−^ cells were gated.(A) CD4^+^CD40L^+^ but not CD4^+^CD40L^−^ T cells express IFN-γ, IL-2, TNF-α, IL-17 or IL-22. The numbers in the quadrants are the percentages of cells. (B) Ratio of IFN-γ, IL-2, TNF-α, (C) IL-17 and IL-22 expression by CD4^+^CD40L^+^ or CD4^+^CD40L^−^ cells. Results shown are the mean ± SEM from 12 to 21 independent experiments. *P<0.05; **P<0.01; ***P<0.001. (D) Histogram graph of T-bet expression by CD4^+^CD40L^+^ T cells. Filled histogram represents isotype control staining and open histogram represents T-bet staining. Data shown are representative of five independent experiments.

To confirm that CD40L identifies *MTB*-specific CD4^+^T cells, we assessed the cytokine production by CD4^+^CD40L^+^ and CD4^+^CD40L^−^ T cells. From the CD4^+^ T cells analyzed, only the cells expressing CD40L produced IFN-γ, IL-2, TNF-α, IL-17 or IL-22 (Fig. 2A), suggesting that the expression of CD40L identifies the majority of the responding Th cells. Moreover, considerably higher levels of Th1 cytokines (IFN-γ, IL-2 and TNF-α) were found to be expressed by CD4^+^CD40L^+^ T cells, suggesting that the majority of CD4^+^CD40L^+^ T cells are Th1 cells. We confirmed this idea by detecting the expression of T-bet, an essential transcriptional factor in Th1 cells. We found that more than 90% of CD4^+^CD40L^+^ T cells express T-bet (Fig. 2D). We also measured the expression of Th2 cytokines and found that little to no IL-4 or IL-10 is induced following stimulation with ESAT-6/CFP-10 peptides (data not shown). We found that Th1 cytokine-producing CD4^+^CD40L^+^ cells were significantly more abundant than CD4^+^CD40L^−^ cells (Fig. 2B). Moreover, CD4^+^CD40L^+^ T cells were found to produce significantly higher amounts of IL-17 and IL-22 than CD4^+^CD40L^−^ T cells. However, the cytokine level was considerably lower than in Th1-cytokine-producing cells (Fig. 2C), suggesting that natural *MTB* infection induces a mixed Th-cell response characterized by Th1, Th17 and Th22 cells.

### CD4^+^CD40L^+^ T cells are dominated by polyfunctional Th1 cells

**Figure 3:**
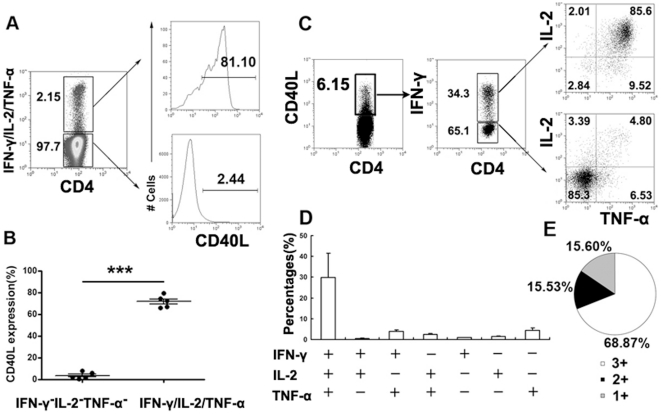
CD4^+^CD40L^+^ T cells express polyfunctional cytokines. PFCs were stimulated with ESAT-6/CFP-10 peptides, anti-CD28 and anti-CD49d mAbs for eight hours. For detection of intracellular cytokines, anti-IFN-γ, anti-IL-2 and anti-TNF-α monoclonal antibodies were labeled with the same fluorescence. The staining of CD40L and cytokines of IFN-γ/IL-2/TNF-α were conducted in one FACS tube.(A) Th1 cytokine (IFN-γ, IL-2 or TNF-α) producing and nonproducing cells were gated. The expression of CD40L was evaluated. (B) Summary of the CD40L expression data within Th1 cytokine producing and nonproducing cells. Horizontal lines represent mean ± SEM. (C) CD4^+^IFN-γ^+^ and CD4^+^IFN-γ^−^ cells within CD4^+^CD40L^+^ T cells were further gated, and the expression of IL-2 and TNF-α are shown. (D) The total antigen response within CD4^+^CD40L^+^ T cells was defined as the number of cells expressing any combination of IFN-γ, IL-2 or TNF-α. The average percentages for each subset are shown. (E) The percentages of cells producing three cytokines (triple positive), two cytokines (double positive) or only one cytokine (single positive) within the total CD4^+^CD40L^+^ T cell response. Data shown are the mean values from five independent experiments.

We next analyzed the expression of CD40L by the Th1 cytokine (IFN-γ, IL-2 or TNF-α)-producing cells and the non-Th1 cytokine-producing CD4^+^ T cells. We found that the CD4^+^ T cells that express at least one Th1 cytokine (IFN-γ, IL-2 or TNF-α) are mostly CD40L^+^ (71.64±2.44%, mean ± SEM); cells that do not express any of the measured Th1 cytokines are mostly CD40L^−^ (3.50±1.39%, Fig. 3A, B). We then analyzed the cytokine profiles of CD4^+^CD40L^+^ T cells by measuring the production of IFN-γ, IL-2 and TNF-α. We found that CD4^+^CD40L^+^ T cells produce IFN-γ, IL-2 and TNF-α, either concurrently or individually (Fig. 3C). We defined the total Th1 response in the CD4^+^CD40L^+^ T cells as the percentage of T cells expressing any combination of IFN-γ, IL-2 or TNF-α. With this metric, seven distinct functional populations could be delineated: IFN-γ/IL-2/TNF-α triple expressers, IFN-γ/IL-2, IFN-γ/TNF-α or TNF-α/IL-2 double expressers or IFN-γ, IL-2 or TNF-α single expressers. Polyfunctional T cells that simultaneously produce IFN-γ, IL-2 and TNF-α (IFN-γ/IL-2/TNF-α triple expressers) were found to dominate the CD4^+^CD40L^+^ T cells. The percentage of polyfunctional T cells was found to be considerably higher than that of the other six subsets (Fig. 3D). The percentage of double producers (15.53%) was found to be similar to the percentage of single producers (15.60%, Fig. 3E).

### CD4+CD40L+ T cells are mostly effector or effector memory T cells

**Figure 4:**
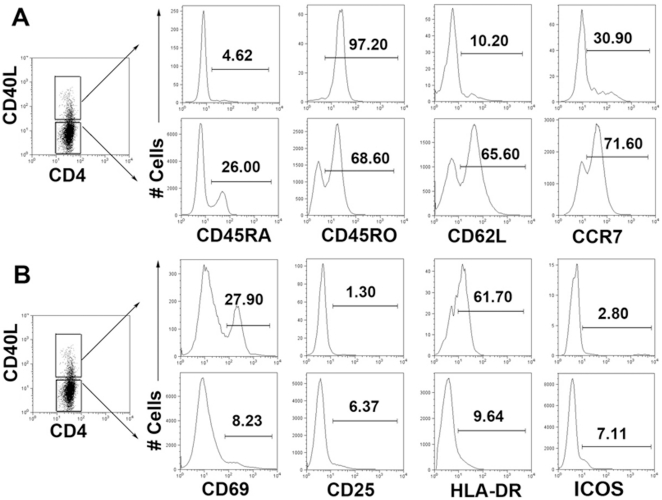
CD4^+^CD40L^+^ and CD4^+^CD40L^−^T cells display different phenotypes. PFCs were stimulated with ESAT-6/CFP-10 peptides, anti-CD28 and anti-CD49d mAbs. CD4^+^CD40L^+^ and CD4^+^CD40L^−^ T cells were gated. (A) The expression of CD45RA, CD45RO, CD62L, CCR7 and (B) the expression of activation markers CD69, CD25, HLA-DR and ICOS by CD4^+^CD40L^+^ and CD4^+^CD40L^−^ T cells. One representative datapoint from five independent experiments is shown.

We next investigated the phenotype of CD4^+^CD40L^+^ T cells with respect to naïve/memory and activation markers. As shown in Fig. 4A, CD4^+^CD40L^+^ T cells were found to be predominantly CD45RO^+^CD45RA^−^, which are effector or memory cells. In contrast, CD4^+^CD40L^−^ T cells contain a significantly higher percentage of CD45RA^+^ cells and a lower percentage of CD45RO^+^ cells. We also found that CD4^+^CD40L^+^ T cells express significantly lower levels of CD62L and CCR7 than CD4^+^CD40L^−^ T cells (Fig. 4A). Because the expression of CD40L was found to be induced following the activation of CD4^+^ T cells, we also looked for the expression of other activation markers, including CD69, CD25, HLA-DR and the inducible costimulator (ICOS). A fraction of CD4^+^CD40L^+^ T cells were found to express CD69 and HLA-DR but not CD25. ICOS was found to be expressed in a small amount of CD4^+^CD40L^−^ T cells but not in CD4^+^CD40L^+^ cells (Fig. 4B).

### The expression of CD40L identifies viable MTB-specific Th cells

**Figure 5:**
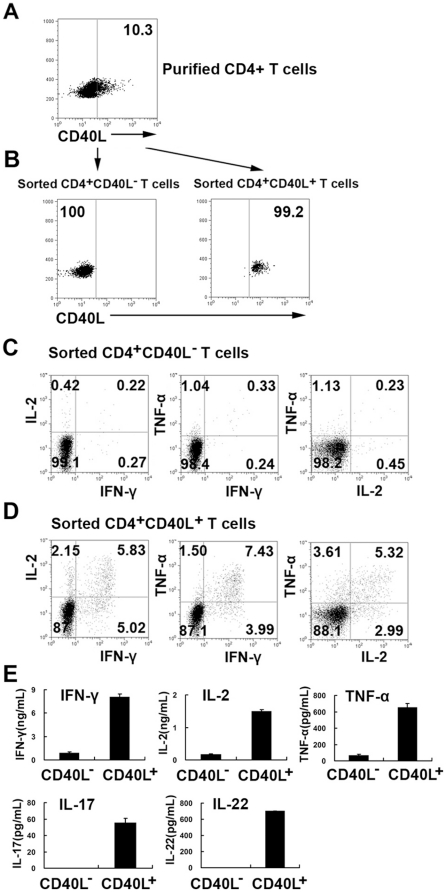
Isolation of viable CD4^+^CD40L^+^ and CD4^+^CD40L^−^ T cells. PFCs were stimulated with ESAT-6/CFP-10 peptides, anti-CD28 and anti-CD49d mAbs in the presence of a fluorescently labeled anti-CD40L monoclonal antibody and monensin. CD4^+^T cells were first isolated with magnetic-beads. (A) The expression of CD40L within purified CD4^+^ T cells was demonstrated, and (B) CD4^+^CD40L^+^ and CD4^+^CD40L^−^ T cells were further sorted by flow cytometry. (C) Sorted CD4^+^CD40L^+^ or (D) CD4^+^CD40L^−^ T cells were co-cultured with purified CD14 cells independently in the presence of ESAT-6/CFP-10 peptides. The intracellular expression of IFN-γ, IL-2 or TNF-α by sorted CD4^+^CD40L^+^ and CD4^+^CD40L^−^ T cells was detected by flow cytometry. (E) The amounts of IFN-γ, IL-2, TNF-α, IL-17 and IL-22 were measured supernatants were measured using ELISA.

We further investigated whether it would be possible to obtain viable purified CD4^+^CD40L^+^ T cells after incubation with *MTB*-specific peptides and a fluorescently labeled anti-CD40L monoclonal antibody and cell sorting with FACS. PFCs were stimulated with ESAT-6/CFP-10 peptides in the presence of a fluorescently labeled CD40L monoclonal antibody and monensin. CD4^+^ T cells and CD14^+^ cells were purified with a positive microbead selection (Fig. 5A). CD4^+^CD40L^+^ and CD4^+^CD40L^−^ T cells were then isolated using flow cytometry (Fig. 5B). We then restimulated either CD4^+^CD40L^+^ or CD4^+^CD40L^−^ T cells with CD14^+^ cells (antigen-presenting cells) in the presence of ESAT-6/CFP-10 peptides and measured the production of cytokines. Notably, CD4^+^CD40L^+^ but not CD4^+^CD40L^−^ fractions produced considerably higher levels of IFN-γ, IL-2 and TNF-α (Fig. 5C, D). Similarly, significantly higher levels of IFN-γ, IL-2 and TNF-α were detected in the supernatants of CD4^+^CD40L^+^ fractions than in those of CD4^+^CD40L^−^ (Fig. 5E). We also assessed the levels of IL-4, IL-10, IL-17 and IL-22 in culture supernatants. Although low levels of IL-17 and IL-22 were detected, it is clear that the CD4^+^CD40L^+^ fractions were highly enriched for both IL-17 and IL-22 compared with CD4^+^CD40L^−^ fractions (Fig. 5E). IL-21, as well as the Th2 cytokines IL-4 and IL-10, was not detectable in our co-culture system (data not shown). Thus, the expression of CD40L can be used to identify the vast majority of *MTB*-specific Th1, Th17 and Th22 cells.

## Discussion

There is clinical and immunological data to suggest that TBP is a good model for understanding protective immune mechanisms against *MTB* at the site of infection [23]. It is well known that T helper (Th) cells that target *MTB* are critical in TB prognosis [24]. Although it is well recognized that Th1 cells are essential in the cell-mediated response to *MTB*, other T helper subsets also participate in the cellular immune responses to *MTB* [11], [25]. Understanding the roles for these Th cell subsets will be essential for the evaluation of *MTB*-specific cellular immune responses and for the immunodiagnosis of TB.

T cell IFN-γ release assays (TIGRA) are more specific and sensitive diagnostic tools for *MTB* infection than the tuberculin skin test (TST) [26], [27], [28]. TIGRA measures IFN-γ production by mononuclear cells following stimulation with restricted antigens that are absent from BCG or most non-tuberculous Mycobacteria (NTM) [1]. Thus, TIGRA cannot be used to measure *MTB*-specific Th cells that do not produce IFN-γ following antigen stimulation.

The complexity of the T helper subsets that are involved in natural TB infection and the limitations of measuring antigen-specific Th cells with cytokine production make the identification of a marker to directly evaluate the entire repertoire of antigen-specific Th cells an important research topic. In this study, we found that CD40L expression on CD4^+^ T cells is a good marker for the evaluation of *MTB*-specific Th cells. This measurement allows us to estimate the quantity, as well as quality, of *MTB*-specific Th cells. We used intracellular staining to detect CD40L expression with BFA [16], and we isolated viable CD4^+^CD40L^+^ cells using a fluorescently-labeled CD40L-specific antibody and monensin treatment [17].

We showed that CD4^+^CD40L^+^ but not CD4^+^CD40L^−^ T cells primarily express IFN-γ, IL-2, TNF-α, IL-17 or IL-22 following stimulation with *MTB*-specific ESAT-6/CFP-10 peptides. The majority of CD4^+^CD40L^+^ T cells were found to be Th1 cells that express IFN-γ, IL-2, TNF-α and T-bet. Although the percentages of IL-17 or IL-22-producing cells were relatively low as compared with those that produce Th1 cytokines, CD4^+^CD40L^+^ cells were found to highly express IL-17 and IL-22 compared with CD4^+^CD40L^−^ T cells. In addition, we also assessed the expression of CD40L and IFN-γ by PFCs following stimulation with non-MTB-specific antigen SARS-CoV S peptide 50 (STFFSTFKCYGVSATKL) or SARS-CoV S peptide 107 (NFSQILPDPLKPTKRSFI) and found that CD4^+^ T cells did not express either IFN-γ or CD40L in response to SARS-CoV peptide. However, PFCs from the same patients expressed CD40L and IFN-γ following stimulation with MTB-specific peptides of ESAT-6/CFP-10. Therefore, we showed that *MTB*-specific Th cells are primarily enriched with CD4^+^CD40L^+^ cells and that CD40L can be a good marker to evaluate *MTB*-specific Th cells, irrespective of the cytokine profile.

A number of studies in humans have shown that polyfunctional T cells that secrete multiple cytokines may indeed mediate protection against TB [29], [30], [31], [32], [33]. Importantly, we confirmed that CD4^+^CD40L^+^ cells are primarily dominated by polyfunctional Th1 cells that can secrete three cytokines simultaneously. Therefore, we believe that CD40L can be used not only as a marker for *MTB*-specific Th cells but also as a highly specific marker of polyfunctional Th cells. In other words, the detection of CD4^+^CD40L^+^ cells could represent *MTB*-specific Th1 cells, and we could thus evaluate polyfunctional T cells without measuring all three cytokines simultaneously. CD4^+^CD40L^+^ T cells display CD45RA^−^CD45RO^+^CCR7^−^CD62L^−^ICOS^−^ effector or effector memory cells with the expression of some activation markers, including CD69 and HLA-DR but not CD25 and ICOS. Our results suggest that CD40L cannot be replaced by other activation markers to evaluate antigen-specific CD4^+^ T cells. Moreover, these markers lack specificity, as they are also known to be expressed by specialized T cell subsets (regulatory T cells) and induced by cytokines [34], [35], [36].

Lastly, we incubated PFCs with ESAT-6/CFP-10 peptides and fluorescently labeled anti-CD40L antibody and sorted CD4^+^CD40L^+^ and CD4^+^CD40L^−^ T cells by flow cytometry. We showed that sorted CD4^+^CD40L^+^ but not CD4^+^CD40L^−^ fractions produce high levels of IFN-γ, IL-2 and TNF-α following restimulation with ESAT-6/CFP-10 peptides. This result is consistent with those of our initial assay. We also demonstrated that sorted CD4^+^CD40L^+^ fractions produce significantly higher levels of IFN-γ, IL-2, TNF-α, IL-17 and IL-22 but not Th2 cytokines (IL-4 and IL-10) compared with CD4^+^CD40L^−^ fractions. These results demonstrate that the expression of CD40L on *MTB*-specific CD4^+^ T cells is a good marker for the evaluation of all T helper subsets involved in TB infection. Our results further suggest that distinct T helper cells are induced during natural *MTB* infection and that these cells differ in their ability to provide CD40L-mediated help from APC and B cells [10].

In conclusion, we showed that CD40L is more specific and sensitive for the analysis and isolation of *MTB*-specific Th cells than any other cytokine or activation molecule and CD40L expression could be a valuable marker for the diagnosis of TB.

## Materials and Methods

### Study participants

A total of twenty-two patients with tuberculous pleurisy (10 females and 12 males, 23–71 years old) were recruited from the Chest Hospital of Guangzhou, China. The diagnosis of pleural effusion from TB etiology was based on the following criteria: (i) *M. tuberculosis* on a pleural fluid smear (by Ziehl-Neelsen method); (ii) pleural fluid or pleural biopsy specimens growing *M. tuberculosis* on Lowenstein-Jensen medium; (iii) histological evidence of caseating granuloma on biopsy specimens of pleural tissue with positive staining for *M. tuberculosis*. The patients who had been diagnosed with HIV, HBV, or HCV or that had a history of autoimmune diseases were excluded from the study. Five healthy volunteers were recruited from Sun Yat-sen University. Written informed consent was obtained from all of the patients and healthy donors. The study was approved by the Zhongshan School of Medicine Review Board (Guangzhou, China).

### Peptides, reagents and mAbs

To detect *M. tuberculosis*-specific responses, we selected six highly immunogenic and largely HLA-DR-restricted peptides. Four were derived from ESAT-6 and two from the CFP-10 protein of *M. tuberculosis*. Synthetic peptides of 20 amino acids (aa) in length were obtained from Shenzhen Hanyu manufacture, Shenzhen, China. The sequences of the peptides are as follows: P1, ESAT-6_1–20_ (MTEQQWNFAGIEAAASAIQG); P2, ESAT-6_31–50_ (EGKQSLTKLAA AWGGSGSEA); P3, ESAT-6_61–80_ (TATELNNALQNLARTISEAG); P4, ESAT-6_71–90_ (NLARTISEAGQAMASTEGNV); P5, CFP-10_51–70_ (AQAAVVRFQEAANKQKQ ELD) and P6, CFP-10_71–90_ (EISTNIRQAGVQYSRADEEQ). Purified anti-CD28 (clone CD28.2) and anti-CD49d (clone 9F10) mAbs were purchased from BD Biosciences (San Jose, CA). The following mAbs were used for phenotypic, intracellular cytokine or transcription factor analyses: CD62L-PE (Dreg56), CCR7-PE (3D12), TNF-α-Pecy7 (MAb11), CD69-Pecy7 (FN50), CD45RA-FITC (L48), CD45RO-FITC (UCHL1), CD25-FITC (M-A251), HLA-DR-PE (L243 (G46-6)), T-bet-PE (O4-46), CD4-PerCP (L200), APC-IL-2 (MQ1-17H12). Isotype-matched control antibodies were purchased from BD Biosciences (San Jose, CA, USA). CD27-APC (O323) was obtained from Biolegend (San Diego, CA). IFN-γ-FITC (45.15) was bought from Beckman Coulter (Fullerton, CA). IL-17A-PE (eBio64CAP17) was purchased from eBioscience (Santiago, USA), and IL-22-PE (142928) was purchased from R & D Systems (Minneapolis, MN). Purified anti-CD3 and anti-CD28 mAbs were purchased from BD Bioscience.

### Preparation of PFCs and PBMCs

PFCs were isolated by first lysing erythrocytes with an ammonium chloride solution and resuspending the pellet to a final concentration of 2×10^6^ cells/mL in complete RPMI 1640 medium (Invitrogen, Grand Island, NY) supplemented with 10% heat-inactivated fetal calf serum (FCS; HyClone, Logan, UT), 100 U/mL penicillin, 100 µg/mL streptomycin, 2 mM L-glutamine, and 50 µM 2-mercaptoethanol. Peripheral blood mononuclear cells (PBMCs) were isolated by Ficoll-Hypaque gradient centrifugation of heparinized venous blood obtained from healthy individuals or TB patients.

### Flow cytometry

To detect intracellular cytokines and CD40L, cells were incubated with 1 µg/mL peptides plus 1 µg/mL anti-CD28 and 1 µg/mL anti-CD49d for 8 h in the presence of brefeldin A (BFA, 10 µg/mL; Sigma-Aldrich, St Louis, MO). After stimulation, cells were washed twice with PBS buffer containing 0.1% BSA and 0.05% sodium azide. Thereafter, cells were incubated with the monoclonal Abs (mAbs) for surface markers at 4°C in the dark for 30 min. The cells were then washed twice and fixed in 4% paraformaldehyde, followed by permeabilization and stained for the intracellular cytokines and CD40L in PBS buffer containing 0.1% saponin. For the analysis of CD40L expression and Th1 cytokine-producing cells, we chose anti-IFN-γ, anti-IL-2 and anti-TNF-α monoclonal antibodies labeled with the same fluorescence. Thereafter, we performed the staining of CD40L and IFN-γ/IL-2/TNF-α in one FACS tube. We could thus analyze CD40L expression and IFN-γ/IL-2/TNF-α expression in one graph. Flow cytometry was performed using a FACSAria ⨿ (BD Biosciences). Data were analyzed using FlowJo software (TreeStar, San Carlos, CA).

### Isolation of cell subsets

PFCs were stimulated with peptides in the presence of PE-labeled anti-CD40L monoclonal antibody and Protein Transport Inhibitor monensin (BD Biosciences Pharmingen) for eight hours. Thereafter, CD4^+^ and CD14^+^ cells were isolated from PFCs by positive selection using anti-CD4 or anti-CD14 microbeads (Miltenyi Biotec, Bergisch Gladbach, Germany). The isolation of CD4^+^CD40L^+^ and CD4^+^CD40L^−^ cells was performed using purified CD4^+^ T cells on a FACSAria ⨿ high-speed cell sorter. The purity of cells, as assessed by flow cytometry, exceeded 97% for each cell subset. Purified CD4^+^CD40L^+^ or CD4^+^CD40L^−^ T cells were co-cultured with purified CD14^+^ cells (at ratio of 1∶2) in the presence of ESAT-6 and CFP-10 peptides. Cell-free supernatants were collected, and IFN-γ, IL-2, TNF-α, IL-4, IL-10, IL-17 and IL-22 were detected with ELISA. The ELISA kit for the detection of IFN-γ, IL-2, TNF-α, IL-4 and IL-10 was purchased from BD Bioscience Pharmingen. The ELISA kit for IL-22 was purchased from R & D Systems. The IL-17 ELISA kit was purchased from eBioscience. To detect intracellular cytokines, cells were cultured for 8 hours, and then flow cytometry was performed.

### Statistical analysis

Wilcoxon matched pairs test (Two-tailed) was used to determine the statistical differences between the groups using GraphPad Prism software version 5. A value of *p*<0.05 was considered statistically significant.
